# Experimental Validation of Multi-Epitope Peptides Including Promising MHC Class I- and II-Restricted Epitopes of Four Known *Leishmania infantum* Proteins

**DOI:** 10.3389/fimmu.2014.00268

**Published:** 2014-06-10

**Authors:** Maria Agallou, Evita Athanasiou, Olga Koutsoni, Eleni Dotsika, Evdokia Karagouni

**Affiliations:** ^1^Laboratory of Cellular Immunology, Department of Microbiology, Hellenic Pasteur Institute, Athens, Greece

**Keywords:** *in silico* analysis, cysteine peptidase A, histone H1, kinetoplastid membrane protein 11, *Leishmania* eukaryotic initiation factor, lymphocyte proliferation, CD8^+^IFN-γ^+^ T cells, CD4^+^IFN-γ^+^ T cells

## Abstract

Leishmaniasis is a significant worldwide health problem for which no vaccine exists. Activation of CD4^+^ and CD8^+^ T cells is crucial for the generation of protective immunity against parasite. Recent trend in vaccine design has been shifted to epitope-based vaccines that are more specific, safe, and easy to produce. In the present study, four known antigenic *Leishmania infantum* proteins, cysteine peptidase A (CPA), histone H1, KMP-11, and *Leishmania* eukaryotic initiation factor (LeIF) were analyzed for the prediction of binding epitopes to H2^d^ MHC class I and II molecules, using online available algorithms. Based on *in silico* analysis, eight peptides including highly scored MHC class I- and II-restricted epitopes were synthesized. Peptide immunogenicity was validated in MHC compatible BALB/c mice immunized with each synthetic peptide emulsified in complete Freund’s adjuvant/incomplete Freund’s adjuvant. CPA_p2, CPA_p3, H1_p1, and LeIF_p6 induced strong spleen cell proliferation upon *in vitro* peptide re-stimulation. In addition, the majority of the peptides, except of LeIF_p1 and KMP-11_p1, induced IFN-γ secretion, while KMP-11_p1 indicated a suppressive effect on IL-10 production. CPA_p2, CPA_p3, LeIF_p3, and LeIF_p6 induced IFN-γ-producing CD4^+^ T cells indicating a T_H1_-type response. In addition, CPA_p2, CPA_p3, and H1_p1 induced also the induction of CD8^+^ T cells. The induction of peptide-specific IgG in immunized mice designated also the existence of B cell epitopes in peptide sequences. Combining immunoinformatic tools and experimental validation, we demonstrated that CPA_p2, CPA_p3, H1_p1, H1_p3, CPA_p2, LeIF_p3, and LeIF_p6 are likely to include potential epitopes for the induction of protective cytotoxic and/or T_H1_-type immune responses supporting the feasibility of peptide-based vaccine development for leishmaniasis.

## Introduction

Leishmaniasis, a vector-borne parasitic disease, is caused by dimorphic protozoan flagellates of the genus *Leishmania* with a worldwide distribution. The disease is characterized by diversity and complexity, presenting a wide spectrum of clinical forms in humans ranging from self-healing cutaneous leishmaniasis (CL) to fatal visceral leishmaniasis (VL). In VL, parasites colonize internal organs, primarily the spleen, liver, and bone marrow. With an estimated 0.5 million cases per year, VL has emerged as an important public-health concern with major clinical and socioeconomic impacts^1^. In South Europe, VL is caused almost exclusively by *Leishmania (L.) infantum*, which is transmitted as a zoonosis with the domestic dog serving as the main reservoir of the parasite (1). Current attempts against leishmaniasis are based on chemotherapy to alleviate disease (2, 3) and on vector control to reduce transmission (4). Toxic side effects and growing resistance to available therapeutic drugs against VL has made the global demand for an effective vaccine capable to elicit a protective immune response, a major public-health priority.

Despite the substantial knowledge regarding the various life stages of the parasite, the considerable inter-specific diversity, the extraordinary host evasive mechanisms of parasite and the heterogeneity of population (5, 6), effective vaccine development against human VL represents an unprecedented challenge. Although a great number of potent vaccine candidates has shown promising results in mice (7, 8) and dogs (9–11), none of them has entered human trials except for LeishF1 with reported phase I and II clinical trials (12, 13).

Antigen identification is considered as a significant barrier in vaccine design, as this is usually achieved through time consuming and labor-intensive *in vitro* and *in vivo* experiments. Efforts have thus focused on developing novel strategies for more rational and faster antigen identification among large numbers of pathogen proteins. Furthermore, recent reports support that epitope-based vaccines appear to be capable of inducing more potent responses than whole protein vaccines (14). Until recently, the search of immunodominant peptides relied on the direct testing of overlapping peptides or peptide libraries. T cell epitope prediction by bioinformatic analysis of protein sequences has been proposed as a promising strategy for vaccine development and an increasing number of tools have been developed, based on different algorithms and methods (15, 16). There is great possibility of missing the emergence of the sequence mutants that would potentially escape the vaccine’s protective effect. Moreover, the fact that T cells from genetically distinct populations would recognize and respond to a single peptide epitope, underline the need of identifying one or more epitope(s) that bind to multiple HLA alleles and cover close to 100% of the genetically diverse human population (17). Multi-peptide-based vaccines are designed to generate a diverse immune response to incorporate antigens and to reduce limitations due to MHC restriction into a single entity.

The effectiveness of a vaccine depends on its capacity to ensure long-lasting cell-mediated immunity. In VL, there is evidence that an interplay of T helper cytokines (T_H1_/T_H2_) is observed, while resistance or resolution of infection is associated with dominant T_H1_ response and CD8^+^ T cells (18–20). Furthermore, successful treatment of VL with sodium stibogluconate requires the presence of both CD4^+^ and CD8^+^ T cells (21) accompanied with IL-12 and IFN-γ production (22). In contrast, T_H2_ response with IL-4 and IL-10 production results in susceptibility to infection and development of severe disease. Murine models of leishmaniasis have been extensively used to study the pathogenesis of the disease and to test novel therapeutic agents or potent vaccine candidates in pre-clinical studies. One of the most widely studied and commonly used model of VL is the BALB/c strain of mice infected intravenously with *L. infantum*. Although this strain is considered to be susceptible and the infection progresses during the first month, the infection is then controlled by the host immune response. This mouse model is comparable to self-controlled oligosymptomatic cases and therefore is useful for the study of the protective immune response (23).

Several reports demonstrate that different leishmanial antigens elicit desired T_H1_ and CTL responses capable to sustain protection against experimental challenges (24). Among these antigens, cysteine peptidase A (CPA), histone H1, kinetoplastid membrane protein 11 (KMP-11), and *Leishmania* eukaryotic initiation factor (LeIF) are considered important immunogens, as supported by numerous studies. Specifically, CPA induces protection against *L. major* in the experimental model of CL through development of specific T_H1_ immune responses (25–27). Histone H1 and KMP-11, structural highly conserved proteins, are able to trigger specific immune responses (28–33), and immunization with these proteins confer protection against *L. major* or *L. infantum* infections in experimental animal models (34–38). Moreover, LeIF, originally described as a T_H1_-type natural adjuvant, is capable of stimulating IL-12 mediated T_H1_ responses in PBMCs of patients (39). Furthermore, recombinant forms of CPA, histone H1, and KMP-11 act as potent B cell immunogens since they are recognized by sera from either recovered or active cases of CL and VL, as well as by sera from asymptomatic or symptomatic dogs with leishmaniasis (40–45).

In the present study, we applied immunoinformatics using currently available online algorithms in order to identify potentially immunogenic T cell epitopes from the above mentioned *L. infantum* proteins, and design multi-epitope peptides containing both MHC class I and II-restricted epitopes as possible candidate peptide vaccines for VL. Immunogenicity of the synthetic multi-epitope peptides in terms of T cell activation was validated in immunized BALB/c mice by analyzing peptide-specific proliferative responses and cytokine production by CD8^+^ and CD4^+^ T cells.

## Materials and Methods

### Protein sequence retrieval and prediction of MHC class I and II binding epitopes

Full protein sequences of selected proteins, CPA, Histone H1, KMP-11, and LeIF, were retrieved from GenBank data^2^ on JPCM5 strain (MCAN/ES/98/LLm-887) and analyzed by SignalP^3^ for the prediction of signal peptides and transmembrane domains (Table 1). Potential MHC class I and II binding epitopes derived from the four *L. infantum* proteins, were predicted by *in silico* analysis, using three online available, binding algorithms named SYFPEITHI^4^, BIMAS^5^, and NetMHCII^6^. The cut-off score was adjusted to ≥18 for SYFPEITHI, ≥100 for BIMAS, and a default prediction threshold (binding affinity <500 nM) depicting accuracy >85% was used for NetMHCII (Tables 2 and 3).

**Table 1:** ***L. infantum* proteins selected as candidate antigens for epitope mapping**.

Protein abbreviation	Protein name	Protein length (aa)/mass (kDa)	GenBank accession number
CPA	Cysteine peptidase A	354/39.0	CAM67356
Histone H1	Histone H1	111/11.1	ABN54817
KMP-11	Kinetoplastid membrane protein 11	92/11.2	CAA64883
LeIF	*Leishmania* eukaryotic initiation factor	403/45.3	CAM65231

**Table 2:** ***In silico* predicted MHC class I-restricted 9-mer epitopes of *L. infantum* proteins**.

Protein	No. of epitope	Epitope sequence	Score^a^				
			SYFPEITHI		BIMAS		
			H2-K^d^	H2-L^d^	H2-K^d^	H2-L^d^	H2-D^d^
CPA	1	8-FFAIVVTIL-18	23	–	1152	–	–
	2	84-HYDVSGKFA-92	23	–	–	–	–
	3	273-LYFGGVVTL-281	23	–	2400	–	–
	4	254-AYVGKNGPV-262	22	–	1200	–	–
	5	23-SALIAQTPL-31	20	–	–	–	–
	6	102-LYLNPNYYA-110	20	–	120	–	–
	7	334-NYVVTATID-343	20	–	–	–	–
	8	7-FFFAIVVTI-15	–	–	1152	–	–
	9	165-QWALKNHSL-173	–	–	240	–	–
	10	62-RFNAFKQNM-70	–	–	144	–	–
	11	319-GYIRLAMGS-327	–	–	120	–	–
	12	179-QVLVSCDNI-187	–	–	115.20	–	–
	13	68-QNMQTAYFL-76	–	–	115.20	–	–
	14	160-GNIEGQWAL-168	–	–	115.20	–	–
Histone H1	15	46-KKAGAKKAV-54	18	–	–	–	–
	16	2-SSDSAVAAL-10	17	19	–	–	–
KMP-11	17	4-TYEEFSAKL-12	20	–	2400	–	–
	18	47-HYEKFERMI-55	19	–	2000	–	–
	19	72-HFKQFAEL-79	18	–	960	–	–
	20	69-HSEHFKQKF-77	–	18	–	–	–
	21	7-EFSAKLDRL-15	–	–	960	–	–
LeIF	22	385-HYHTQIDEL-393	24	–	2000	–	–
	23	360-RYGRKGVAI-368	23	–	2000	–	–
	24	8-APQDQDSFL-16	–	22	–	225	–
	25	25-IPSFDDMPL-33	–	22	–	150	–
	26	199-LPKDIQVAL-207	–	22	–		–
	27	344-LPTNKENYL-352	–	22	–	150	–
	28	393-LPVDFAAYL-401	–	22	–	150	–
	29	14-SFLDDQPGV-22	21	–	576	–	–
	30	197-RFLPKDIQV-205	21	–	480	–	–
	31	100-LSPTRELAL-108	–	21	–		–
	32	187-GFADQIYEI-195	20	–	960	–	–
	33	318-SRVLVTTDL-326	20	–	–	–	–
	34	23-RPIPSFDDM-31	–	20	–	150	–
	35	123-NSSKFCETF-131	–	20	–	–	–
	36	265-VSIAQSVIF-273	–	20	–	–	–
	37	222-KFMRDPVRI-230	–	–	2304	–	–
	38	195-IFRFLPKDI-203	–	–	960	–	–
	39	272-IFANTRRKV-280	–	–	288	–	–
	40	312-TFRSGSSRV-320	–	–	288	–	–
	41	164-RGALRTESL-172	–	–	–	–	120

*^a^The cut-off score was adjusted to ≥18 for SYFPEITHI, ≥100 for BIMAS, and <500 for NetMHCII*.

**Table 3:** ***In silico* predicted MHC class II-restricted 15-mer epitopes of *L. infantum* proteins**.

Protein	No. of epitope	Epitope sequence	Score^a^		
			SYFPEITHI		NetMHCII
			H2-IA^d^	H2-IE^d^	H2-IA^d^
CPA	1	149-MCGSCWAFATTGNIE-163	28	–	–
	2	246-PHDEEEIAAYVGKNG-260	27	–	–
	3	114-KDYKEHVHVDDSVRS-128	26	–	–
	4	257-GKNGPVAVAVDATTW-271	26	–	–
	5	32-GVDDFIASAHYGRFK-46	25	–	–
	6	312-GSSWGEKGYIRLAMG-326	–	24	–
	7	4-RNPFFFAIVVTILFV-18	23	–	–
	8	5-NPFFFAIVVTILFVV-19	23	–	–
	9	13-VTILFVVCYGSALIA-27	22	–	–
	10	172-SLVSLSEQVLVSCDN-186	22	–	–
	11	174-VSLSEQVLVSCDNID-188	22	–	–
	12	12-VVTILFVVCYGSALI-26	21	–	–
	13	260-GPVAVAVDATTWQLY-274	21	–	–
	14	279-VTLCFGLSLNHGVLV-293	21	–	–
	15	67-KQNMQTAYFLNAHNP-81	20	–	–
	16	273-LYFGGVVTLCFGLSL-287	20	–	–
	17	301-KPPYWIVKNSWGSSW-315	20	–	–
	18	328-NQCLLKNYVVTATID-342	20	–	–
	19	216-SYPYTSAGGTRPPCH-230	–	20	–
	20	308-KNSWGSSWGEKGYIR-322	–	20	–
Histone H1	21	1-MSSDSAVAALSAAMT-15	31	–	88.3
	22	27-KTAAKKAAAKKAAAK-41	29	–	182.6
	23	32-KAAAKKAAAKKAGAK-46	29	–	239.9
	24	37-KAAAKKAGAKKAGAK-51	29	–	–
	25	42-KAGAKKAGAKKAVRK-56	29	–	–
	26	2-SSDSAVAALSAAMTS-16	28	–	123.7
	27	56-KVATPKKPAKKAAKK-70	24	–	–
	28	36-KKAAAKKAGAKKAGA-50	–	24	–
	29	41-KKAGAKKAGAKKAVR-55	–	24	–
	30	71-AAKKPAKKVAKKPAK-85	–	24	–
	31	16-SPQKSPRSSPKKTAA-30	–	22	–
	32	21-PRSSPKKTAAKKAAA-35	–	22	–
	33	26-KKTAAKKAAAKKAAA-40	–	22	163.5
	34	31-KKAAAKKAAAKKAGA-45	–	22	190.3
	35	45-AKKAGAKKAVRKVAT-59	–	22	–
	36	51-KKAVRKVATPKKPAK-65	–	22	–
	37	55-RKVATPKKPAKKAAK-69	–	22	–
	38	59-TPKKPAKKAAKKAAK-73	–	22	–
	39	63-PAKKAAKKAAKKPAK-77	–	22	–
	40	67-AAKKAAKKPAKKVAK-81	–	22	–
	41	75-PAKKVAKKPAKKAAK-89	–	22	–
	42	79-VAKKPAKKAAKKPAK-93	–	22	–
	43	83-PAKKAAKKPAKKPAK-97	–	22	–
	44	87-AAKKPAKKAAKKAAK-101	–	22	–
	45	91-PAKKPAKKAAKKAAK-105	–	22	–
	46	95-PAKKAAKKAAKKAAA-109	–	22	–
	47	5-SAVAALSAAMTSPQK-19	21	–	382.8
	48	76-AKKVAKKPAKKAAKK-90	21	20	–
	49	96-AKKAAKKAAKKAAAK-110	21	–	–
	50	29-AAKKAAAKKAAAKKA-43	–	–	107.9
	51	30-AKKAAAKKAAAKKAG-44	–	–	119.1
	52	25-PKKTAAKKAAAKKAA-39	–	–	119.6
	53	24-SPKKTAAKKAAAKKA-38	–	–	138.5
	54	3-SDSAVAALSAAMTSP-17	–	–	166.2
	55	28-TAAKKAAAKKAAAKK-42	–	–	183.3
	56	35-AKKAAAKKAGAKKAG-49	–	–	208.6
	57	34-AAKKAAAKKAGAKKA-48	–	–	232.0
	58	4-DSAVAALSAAMTSPQ-18	–	–	272.1
	59	48-AGAKKAVRKVATPKK-62	–	–	295.2
	60	33-AAAKKAAAKKAGAKK-47	–	–	317.8
	61	49-GAKKAVRKVATPKKP-63	–	–	457.6
KMP-11	62	4-TYEEFSAKLDRLDEE-18	23	–	–
	63	75-QKFAELLEQQKAAQN-89	19	–	–
	64	45-KEHYEKFERMIKEHT-59	–	18	–
	65	74-KQKFAELLEQQKAAQ-88	–	18	–
LeIF	66	100-LSPTRELALQTAEVI-114	28	–	264.2
	67	320-VLVTTDLVARGICVH-334	28	–	–
	68	199-LPKDIQVALFSATMP-213	27	–	–
	69	387-HTQIDELPVDFAAYL-401	27	–	–
	70	138-QDDLRKLQAGVIVAV-152	26	–	205.3
	71	166-ALRTESLRVLVLDEA-180	26	–	–
	72	169-TESLRVLVLDEADEM-183	26	–	–
	73	62-RGGDIIAQAQSGTGK-76	25	–	–
	74	140-DLRKLQAGVIVAVGT-154	24	–	256.8
	75	142-RKLQAGVIVAVGTPG-156	24	–	–
	76	259-MDLYETVSIAQSVIF-273	24	–	–
	77	223-FMRDPVRILVKRESL-237	–	24	–
	78	325-DLVARGIDVHHVNIV-339	23	–	–
	79	168-RTESLRVLVLDEADE-182	22	–	–
	80	263-ETVSIAQSVIFANTR-277	22	–	–
	81	293-TVSSMHAEMPKSDRE-307	22	–	–
	82	314-RSGSSRVLVTTDLVA-328	22	–	–
	83	268-AQSVIFANTRRKVDW-282	–	22	–
	84	16-LDDQPGVRPIPSFDD-30	20	–	–
	85	71-QSGTGKTGAFSIGLL-85	20	–	–
	86	107-ALQTAEVISRIGEFL-121	20	–	–
	87	174-VLVLDEADEMLSQGF-188	20	–	–
	88	255-LDTLMDLYETVSIAQ-269	20	–	–
	89	376-VELLHEIEAHYHTQI-390	20	–	–
	90	77-TGAFSIGLLQRLDFR-91	–	20	–
	91	81-SIGLLQRLDFRHNLI-95	–	20	–
	92	158-VSDVIKRGALRTESL-172	–	20	–
	93	102-PTRELALQTAEVISR-116	–	–	95.3
	94	103-TRELALQTAEVISRI-117	–	–	95.4
	95	101-SPTRELALQTAEVIS-115	–	–	163.8
	96	139-DDLRKLQAGVIVAVG-153	–	–	249.0
	97	99-VLSPTRELALQTAEV-113	–	–	263.1
	98	98-LVLSPTRELALQTAE-112	–	–	287.6
	99	141-LRKLQAGVIVAVGTP-155	–	–	296.7
	100	49-PSSIQQRAIAPFTRG-63	–	–	320.9
	101	48-KPSSIQQRAIAPFTR-62	–	–	378.0
	102	50-SSIQQRAIAPFTRGG-64	–	–	434.6
	103	97-GLVLSPTRELALQTA-111	–	–	452.0
	104	290-SNHTVSSMHAEMPKS-304	–	–	489.1

*^a^The cut-off score was adjusted to ≥18 for SYFPEITHI, ≥100 for BIMAS, and <500 for NetMHCII*.

### Synthetic multi-epitope peptides

Based on the prediction results of the algorithms used, 9-mer epitopes MHC class I-restricted and 15-mer epitopes MHC class II-restricted giving high score against H2^d^ alleles were extracted and combined in order to generate multi-epitope peptides for each *L. infantum* protein. Thus, 8 peptides, 20–30 amino acid (aa) length, were designed in a way that each peptide included at least one MHC class I-restricted epitope scored very high, as well as adjacent or overlapping MHC class II-restricted epitopes scored also high. Sequence homology between each multi-epitope peptide and mouse proteome were analyzed on BLAST database^7^ and peptides with 100% identity were excluded or re-designed to avoid potential autoimmunity (Table 4). Two multi-epitope peptides of CPA (160–189 and 273–302 aa) and Histone H1 (1–20 and 43–61 aa), one peptide of KMP-11 (4–23 aa), and three peptides of LeIF (6–35, 181–210, and 371–400 aa) were synthesized by GeneCust (Labbx, Dudelange, Luxembourg) with purity ≥95%. Synthetic peptides were dissolved in DMSO, acetic acid (10% in dH_2_O), or dH_2_O according to their hydrophobicity, by vigorous pipetting and stored in aliquots, in −80°C until use. Peptides solutions were found endotoxin free, since LPS concentration was <5 EU/mg as determined by LAL Test Cartridges Portable Test System (Endosafe, Charles River Laboratories, USA). Synthetic multi-epitope peptides were also checked for the presence of 9-mer or/and 15-mer epitopes able to bind to HLA alleles (A2, A3, A24, B7, B15, DP, DQ, DR supertypes) using the above mentioned algorithms (Table 4). In addition, data on the crystal structure of HLA-A2 and HLA-DRB1 molecules were obtained from Protein Data Bank (PDB, codes 1HHG and 2SEB, respectively) and multi-epitope peptides of length 30 aa were transformed into PDB files using SWISS-MODEL, and each of them was docked with HLA-A2 or HLA-DRB1 molecule using the ClusPro program^8^ for structure-based analysis (46–49).

**Table 4:** **Synthetic multi-epitope peptides including MHC class I and II-restricted epitopes**.

Peptide name	Synthetic multi-epitope peptide sequence	Included epitopes	HLA supertype
CPA_p2	160-GNIEGQWALKNHSLVSLSEQVLVSCDNIDD-189	165-QWALKNHSL-173	HLA-A2 (A*0201), HLA-A3 (A*03), HLA-DRB1, HLA-DPA1, HLA-DQA1
		179-QVLVSCDNI-187	
		160-GNIEGQWAL-168	
		172-SLVSLSEQVLVSCDN-186	
		174-VSLSEQVLVSCDNID-188	
CPA_p3	273-LYFGGVVTLCFGLSLNHGVLVVGFNRQAKP-302	273-LYFGGVVTL-281	HLA-A2 (A*0201), HLA-A3 (A*03), HLA-A24 (A*2402), HLA-DRB1, HLA-DPA1, HLA-DQA1
		279-VTLCFGLSLNHGVLV-293	
		273-LYFGGVVTLCFGLSL-287	
H1_p1	1-MSSDSAVAALSAAMTSPQKS-20	2-SSDSAVAAL-10	HLA-A2 (A*0201), HLA-A3 (A*03), HLA-DRB1, HLA-DQA1
		1-MSSDSAVAALSAAMT-15	
		2-SSDSAVAALSAAMTS-16	
		5-SAVAALSAAMTSPQK-19	
H1_p3	43-AGAKKAGAKKAVRKVATPKK-61	46-KKAGAKKAV-54	HLA-A2 (A*0201), HLA-A3 (A*03), HLA-DRB1
		42-KAGAKKAGAKKAVRK-56	
		45-AKKAGAKKAVRKVAT-59	
KMP-11_p1	4-TYEEFSAKLDRLDEEFNRKM-23	4-TYEEFSAKL-12	HLA-A3 (A*03), HLA-A24, HLA-DRB1, HLA-DPA1, HLA-DQA1
		7-EFSAKLDRL-15	
		4-TYEEFSAKLDRLDEE-18	
LeIF_p1	6-KIAPQDQDSFLDDQPGVRPIPSFDDMPLHQ-35	8-APQDQDSFL-16	HLA-B7 (B*5101), HLA-B15 (B62), HLA-DRB1, HLA-DPA1, HLA-DQA1
		25-IPSFDDMPL-33	
		14-SFLDDQPGV-22	
		23-RPIPSFDDM-31	
		16-LDDQPGVRPIPSFDD-30	
LeIF_p3	181-DEMLSQGFADQIYEIFRFLPKDIQVALFSA-210	199-LPKDIQVAL-207	HLA-B7 (B*3501, B*5101), HLA-DRB1, HLA-DPA1, HLA-DQA1
		197-RFLPKDIQV-205	
		187-GFADQIYEI-195	
		195-IFRFLPKDI-203	
		199-LPKDIQVALFSATMP-213	
LeIF_p6	371-VTEKDVELLHEIEAHYHTQIDELPVDFAAY-400	385-HYHTQIDEL-393	HLA-A3 (A*0301), HLA-A24, HLA-DRB1, HLA-DPA1, HLA-DQA1
		393-LPVDFAAYL-401	
		320-VLVTTDLVARGICVH-334	
		387-HTQIDELPVDFAAYL-401	
		376-VELLHEIEAHYHTQI-390	

### Immunization of BALB/c mice

Eight groups of female BALB/c mice (*n* = 8/group), 6–8 weeks old, were immunized subcutaneously at upper and lower dorsal region, with 100 μl emulsion consisting of 50 μg of each synthetic multi-epitope peptide in complete Freund’s adjuvant (CFA). Mice were also received a second immunization with 100 μl emulsion of 50 μg of the same peptide in incomplete Freund’s adjuvant (IFA), as well as a third immunization with 50 μg of peptide alone in PBS at 2 weeks intervals. Two sex and age matched groups of mice (*n* = 8/group) immunized similarly either with the adjuvant or with PBS alone, were served as control groups.

Animals were obtained from the breeding unit of the Hellenic Pasteur Institute (Athens, Greece) and reared in institutional facilities under specific pathogen-free conditions, receiving a diet of commercial food pellets and water *ad libitum*. All experimental procedures had been approved by the institutional Animal Bioethics Committee regulating according to the EU Directive 2010/63 and the National Law 2013/56.

### Culture of lymphocytes and proliferation assays

Fifteen days post the third immunization, spleens from immunized and control mice (*n* = 3/group) were collected in aseptic conditions and used for the preparation of single cell suspensions in RPMI-1640 medium (Biochrom AG, Berlin, Germany) supplemented with 2 mM l-glutamine, 10 mM Hepes, 24 mM NaHCO_3_, 0.05 mM β-mercaptoethanol, 100 U/ml penicillin, 100 μg/ml streptomycin, and 10% (v/v) heat inactivated fetal bovine serum (FBS; Gibco, Paisley, UK) at a density of 1 × 10^6^ cells/ml. Cell viability was >95% as determined by trypan blue exclusion. A volume of 200 μl/well were placed in triplicate into 96-well U-bottomed plates in the presence of various concentrations of each synthetic multi-epitope peptide ranging from 5 to 40 μg/ml and incubated for 96 h in 5% CO_2_ at 37°C in a humidified atmosphere. The optimal concentration for each peptide was determined at 10 μg/ml and used thereafter for recall stimulation. Cells cultured in medium alone or in the presence of Concanavalin A (6 μg/ml) were served as negative or positive control, respectively. Cells were pulsed with 1 μCi/ml of ^3^H-TdR (GE Healthcare, Buckinghamshire, UK) for the final 18 h of the culture period. Cells were harvested and ^3^H-TdR incorporation was determined on a microplate scintillation counter (Microbeta Trilux, Wallac, Turku, Finland). The results were expressed as Δcpm (cpm of cells from immunized mice stimulated with peptide – cpm of immunized mice cultured in medium alone). Proliferative response against to each synthetic peptide giving Δcpm > 2000 was considered as positive.

### Cytokine detection and flow cytometry

Spleen cells from immunized and control mice (*n* = 5/group) were also used for cytokine detection and flow cytometry. Briefly, 1 ml/well of lymphocytes in complete RPMI-1640 medium at a density of 2 × 10^6^ cells/ml were placed in triplicate into 24-well plates and stimulated with 10 μg/ml of each synthetic multi-epitope peptide. Cells were cultured for 72 h in 5% CO_2_ at 37°C in a humidified atmosphere. At the end of the incubation period, culture supernatants were collected and stored at −80°C until analyzed for their cytokine content. The concentrations of IFN-γ and IL-10 in the supernatants were determined by sandwich ELISA kits (900-K98, 900-K53; PeproTech, Rocky Hill, NJ, USA) according to the manufacturer’s instructions. The cytokine concentrations were calculated by reference to standard curves; detection threshold for IFN-γ and IL-10 was 23 and 47 pg/ml, respectively.

In parallel, at 48 h of culture period, similarly cultured cells were exposed for 4 h to 2.5 μg/ml brefeldin A (Fluka, Buchs, Germany), washed in FACS buffer (PBS-2% FBS) and stained with anti-CD4 and anti-CD8 monoclonal antibodies (mAbs) conjugated either with FITC (anti-CD4-FITC, clone RM4-5) or PE (anti-CD4-PE, clone H129.19; anti-CD8-PE, clone 53-6.7) for 30 min. For the identification of intracellular cytokine production, cells were permeabilized using FACS buffer supplemented with 0.1% (v/v) saponin (Sigma) and stained for 30 min on ice with anti-IFN-γ conjugated with FITC (clone XMG1.2) or anti-IL-4 conjugated with PE (clone BVD4-1D11) mAbs. In all cases, control cells were processed similarly using matched isotype control. All mAbs used in the study, were purchased from BD Biosciences (Erembodegem, Belgium). For each sample, 20,000 cells were analyzed on a FACSCalibur (Becton-Dickinson, San Jose, CA, USA) and the data were processed with Cell Quest Software (Becton-Dickinson). The percentage of specific cytokine-producing CD4^+^ or CD8^+^ T cells relative to total numbers of CD4^+^ or CD8^+^ T cells was determined by analysis of FACS data using the FlowJO software package (Tree Star, Inc., Ashland, OR, USA). The percentage of peptide-specific cytokine-producing cells was normalized to their respective proportion in unstimulated cells from mice immunized with CFA/IFA alone, in order to allow for comparison among all the synthetic multi-epitope peptides.

### Enzyme linked immunosorbent assays

Blood collected from each group of mice (*n* = 8/group) at fifteenth day post the third immunization, were centrifuged at 4000 × *g* for 5 min and separated sera were aliquoted for the detection of specific antibodies against each synthetic multi-epitope peptide by specific ELISAs as previously described (50). In brief, 96-well microtiter plates were coated with 5 μg/ml of each individual peptide in carbonate buffer (15 mM Na_2_CO_3_, 35 mM NaHCO_3_), pH 9.6 and left overnight at 4°C. For the detection of total IgG antibodies, 10-fold dilutions of each serum sample in 1% BSA in PBS-T were added and incubated with HRP-labeled goat anti-mouse IgG (1/1000 dilution; GE Healthcare, Buckinghamshire, UK). For the detection of IgG1 and IgG2a isotypes, serum samples (1/100 dilution) were added and plates were similarly incubated either with biotin-labeled rat anti-mouse IgG1 (500 ng/ml; AbD Serotec, Oxford, UK) or IgG2a (250 ng/ml; AbD Serotec) followed by the addition of streptavidin–HRP (1/5000 dilution; AbD Serotec) and incubation for 1 h at 37°C. The cut-off value was determined as the mean OD value of normal mouse serum in a 1/100 dilution + 2SD.

### Statistical analysis

Data were expressed as the mean value with the standard deviation (SD) indicated. Statistical significant differences of the mean values between groups of mice immunized with synthetic multi-epitope peptide emulsified in CFA/IFA and mice immunized with CFA/IFA alone were assessed by unpaired Student’s *t*-test. The probability (*p*) of <0.05 was considered to indicate statistical significance.

## Results

### *In silico* prediction of promising epitopes of *L. infantum* proteins bind to MHC class I and II molecules

CPA, Histone H1, KMP-11, and LeIF have already been defined as candidate antigens. CPA, a protein significantly up-regulated in mature amastigotes (40), is predicted as a secretory protein by SignalP (cleavage site between position 24 and 25 residue), while Histone H1, an also highly expressed protein in mature amastigotes (51), KMP-11, a cytoskeleton-associated protein, and LeIF constitutively expressed in both promastigotes and amastigotes (45, 52), are predicted as non-secretory. *In silico* analysis of proteins for the prediction of binding epitopes to H2^d^ MHC class I and II molecules revealed, in total, 41 9-mer and 104 15-mer peptides, respectively, which scored above the cut-off value of each algorithm used for the prediction (Tables 2 and 3).

In particular, 14 and 20 highly scored binding peptides to H2-K^d^ and H2-IA^d^/IE^d^ alleles, respectively, were predicted by BIMAS and SYFPEITHI for CPA. These peptides were spanning throughout the protein amino-acid sequence and they covered the 69.2% of its length. In addition, 2 and 41 binding peptides to H2-K^d^/L^d^ and H2-IA^d^/IE^d^ alleles, respectively, were predicted by SYFPEITHI and NetMHCII for Histone H1. The peptides were also spanning throughout Histone H1 amino-acid sequence, covering the entire length of the protein. Five and four binding peptides to H2-K^d^/L^d^ and H2-IA^d^/IE^d^ alleles, respectively, were predicted by BIMAS and SYFPEITHI for KMP-11. Peptides were gathered in the middle, as well as in the amino- and carboxy-terminal region of KMP-11 sequence, and covered the 55.4% of its length. In regards to LeIF, 20 and 39 binding peptides to H2-K^d^/L^d^/D^d^ and H2-IA^d^/IE^d^ alleles, respectively, were predicted by BIMAS, SYFPEITHI, and NetMHCII. Peptides were spanning throughout LeIF amino-acid sequence and covered the 79.4% of the entire protein.

### Synthetic multi-epitope peptides containing both MHC class I- and II-restricted epitopes

Based on the above data, eight peptides, 20–30 amino-acid length, were designed and synthesized. At least, one MHC class I-restricted epitope scored very high, as well as adjacent or overlapping MHC class II-restricted epitopes scored also high were nested in each synthetic peptide (Table 4). These multi-epitope peptides included CPA_p2 (160-GNIEGQWALKNHSLVSLSEQVLVSCDNIDD-189) and CPA_p3 (273-LYFGGVVTLCFGLSLNHGVLVVGFNRQAKP-302) from CPA, H1_p1 (1-MSSDSAVAALSAAMTSPQKS-20) and H1_p3 (43-AGAKKAGAKKAVRKVATPKK-61) from Histone H1, KMP-11_p1 (4-TYEEFSAKLDRLDEEFNRKM-23) from KMP-11, LeIF_p1 (6-KIAPQDQDSFLDDQPGVRPIPSFDDMPLHQ-35), LeIF_p3 (181-DEMLSQGFADQIYEIFRFL PKDIQVALFSA-210), and LeIF_p6 (371-VTEKDVELLHEIEAHYHTQIDELPVDFAAY-400) from LeIF. Synthetic peptide sequences showed to be retrieved from highly conserved regions of *L. infantum* proteins, since protein BLAST analysis revealed up to 95% residue identity to homologous sequences of corresponding proteins of strains belonging to *L. major* and *L. donovani* complexes. In addition, promiscuous 9-mer and 15-mer epitopes bound to HLA alleles (A2, A3, A24, B7, B15, DP, DQ, DR supertypes) were also nested in synthetic multi-epitope peptides as predicted by *in silico* analysis using the above mentioned algorithms.

### Validation of synthetic multi-epitope peptides immunogenicity in mice

**Figure 1:**
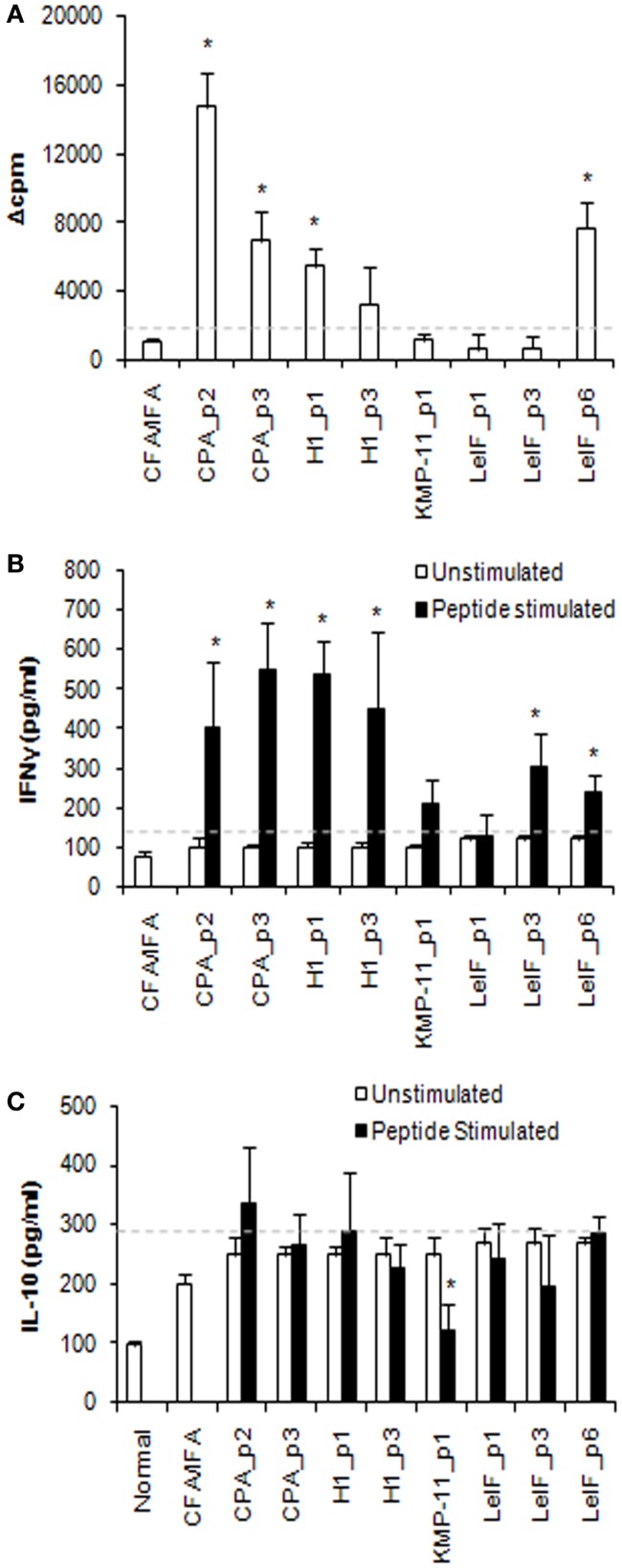
**Multi-epitope peptide-specific proliferative responses and cytokine secretion**. **(A)** Proliferative responses. Spleen cells from BALB/c mice (*n* = 3/group) immunized either with individual peptide emulsified in CFA/IFA or PBS alone, were re-stimulated *in vitro* with the respective peptide (10 μg/ml) for 72 h. Cultures were pulsed for the final 18 h with 1 μCi of [^3^H]-TdR and results are depicted as Δcpm ± SD as described in Section “Materials and Methods.” Spleen cells derived from mice immunized with PBS alone, stimulated *in vitro* with ConA (Δcpm: 39743 ± 843) were used for comparison purposes. **(B)** IFN-γ and **(C)** IL-10 secretion. Cytokines were detected in culture supernatants of spleen cells from immunized BALB/c mice (*n* = 5/group), re-stimulated *in vitro* with the respective peptide (10 μg/ml) for 72 h, by ELISA. The results are expressed as pg/ml ± SD. Significant differences between groups of mice immunized with each synthetic peptide emulsified in CFA/IFA and the group of mice immunized with CFA/IFA alone are indicated by * (*P* < 0.05).

Immunogenicity of the eight synthetic multi-epitope peptides was validated in BALB/c mice (H2^d^ haplotype) immunized with each synthetic peptide in combination with CFA/IFA, 15 days post third immunization. Specific proliferative T cell responses induced by synthetic multi-epitope peptides were firstly assessed. As shown in Figure 1A, CPA_p2, CPA_p3, H1_p1, and LeIF_p6 induced strong proliferation of spleen cells upon *in vitro* re-stimulation (Δcpm > 1128 ± 165) at the optimal dose of 10 μg/ml. Of these, CPA_p2 induced the strongest proliferation, followed by LeIF_p6, CPA_p3, and H1_p1. The results indicated that four of the eight candidate peptides could effectively induce spleen cell proliferation.

To validate the profile of cytokines secreted in response to the eight synthetic multi-epitope peptides, spleen cell culture supernatants from immunized mice were analyzed for their content in IFN-γ and IL-10 at 72 h post respective peptide *in vitro* re-stimulation. Quantitation by ELISA revealed that all peptides, except from LeIF_p1 and KMP-11_p1, induced the secretion of high amounts of IFN-γ in comparison to mice immunized with CFA/IFA alone (Figure 1B). CPA_p3, H1_p1, CPA_p2, and H1_p3 were able to induce the highest secretion of IFN-γ, followed by LeIF_p3 and LeIF_p6. In contrast, unstimulated spleen cells from immunized mice produced low levels of IFN-γ spontaneously, similar to those measured in the culture supernatants of spleen cells from mice immunized with CFA/IFA alone. The results suggest that the majority of the candidate peptides could induce IFN-γ secretion.

In addition, low levels of IL-10 were detected in the supernatants of spleen cells stimulated *in vitro* with each synthetic multi-epitope peptide (Figure 1C). These levels were comparable to those detected in the supernatants of unstimulated spleen cells, as well as in the supernatants of spleen cells from mice immunized with CFA/IFA alone. In particular, KMP-11_p1 indicated a rather suppressive effect on IL-10 production.

**Figure 2:**
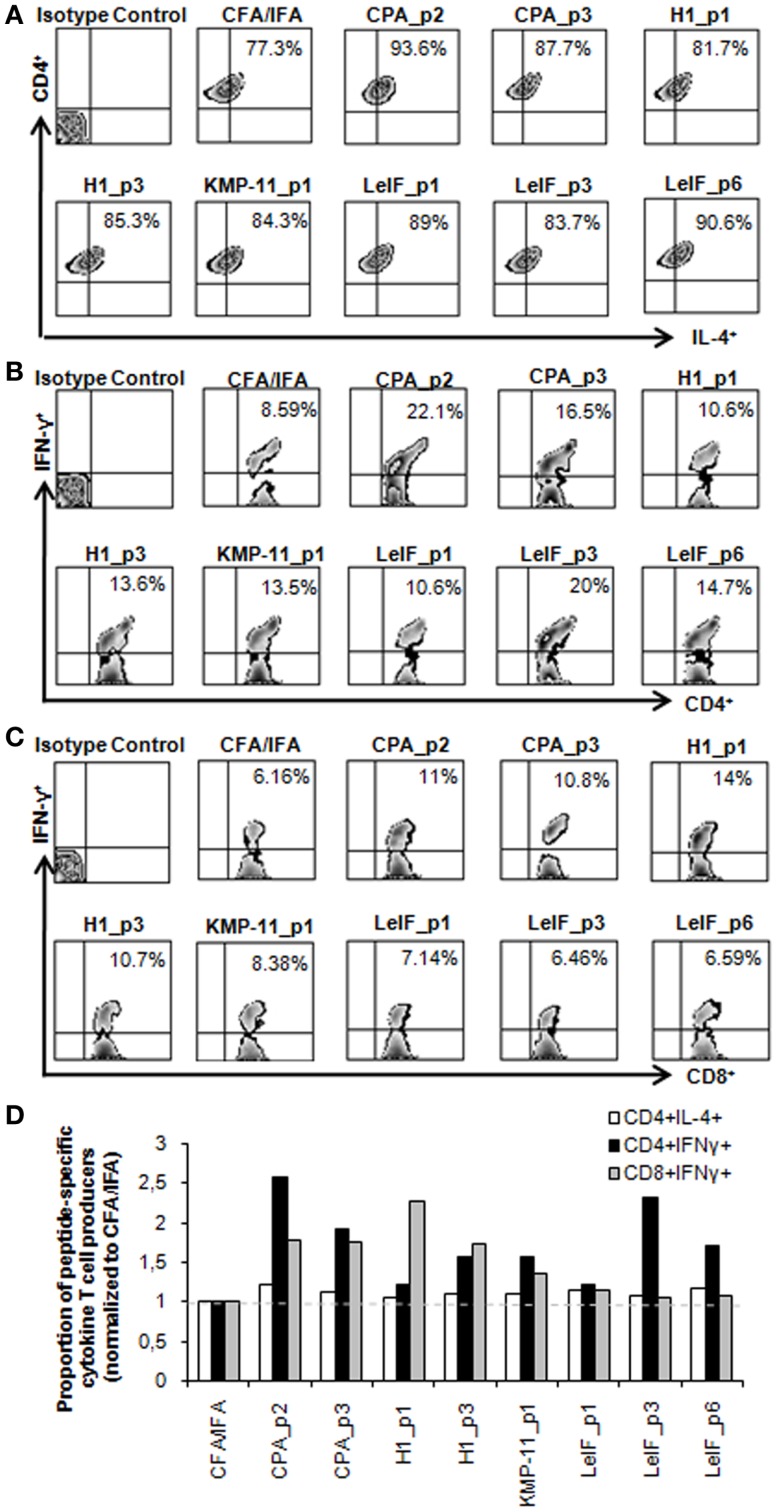
**Multi-epitope peptide-specific cytokine production by CD4^+^ and CD8^+^ T cells**. Spleen cells from BALB/c mice (*n* = 5/group) immunized either with individual peptide emulsified in CFA/IFA or PBS alone, were re-stimulated *in vitro* with the respective peptide (10 μg/ml) for 48 h, and analyzed for CD4^+^ and CD8^+^ T cells producing IL-4 and IFN-γ. **(A–C)** Representative FACS plots of intracellular staining used to define IL-4- and IFN-γ-expressing CD4^+^ and CD8^+^ T cells in spleens derived from immunized mice. Values represent the percentages of **(A)** IL-4^+^ cells among CD4^+^ T cells and **(B,C)** IFN-γ^+^ cells among CD4^+^ and CD8^+^ T cell populations. **(D)** Quantification of IL-4 and IFN-γ producing T cells in immunized mice. All data were normalized to CFA/IFA control group. Each bar represents the mean proportion of IL-4- and IFN-γ-producing CD4^+^ and CD8^+^ T cells induced by the respective peptide.

To further confirm the pattern of cytokines induced by each synthetic multi-epitope peptide, intracellular cytokine production was determined in spleen cells from immunized mice at 48 h post peptide *in vitro* re-stimulation using flow cytometry. As shown in Figure 2A, none of the peptides tested were able to stimulate important peptide-specific IL-4 production by CD4^+^ T cells, although a certain predisposition in BALB/c mice has been documented by previous studies (53). In contrast, CPA_p2, CPA_p3, H1_p3, LeIF_p3, and LeIF_p6 were able to stimulate important peptide-specific IFN-γ production by CD4^+^ T cells, indicating a T_H_ cell driven toward the T_H1_ type.

Regarding the ability of the synthetic multi-epitope peptides to induce the production of IFN-γ by CD8^+^ T cells, flow cytometry revealed that one of them, H1_p1 strongly induced the production of IFN-γ by splenic CD8^+^ T cells of immunized mice. H1_p3, CPA_p2, and CPA_p3 were also able to stimulate peptide-specific IFN-γ production by CD8^+^ T cells in a lower level than that detected in H1_p1 (Figure 2C). Flow cytometry overall results indicated that most of the peptides tested induced IFN-γ production from CD4^+^ and/or CD8^+^ T cells confirming the results obtained with *in silico* analysis (Figures 2B,D).

**Figure 3:**
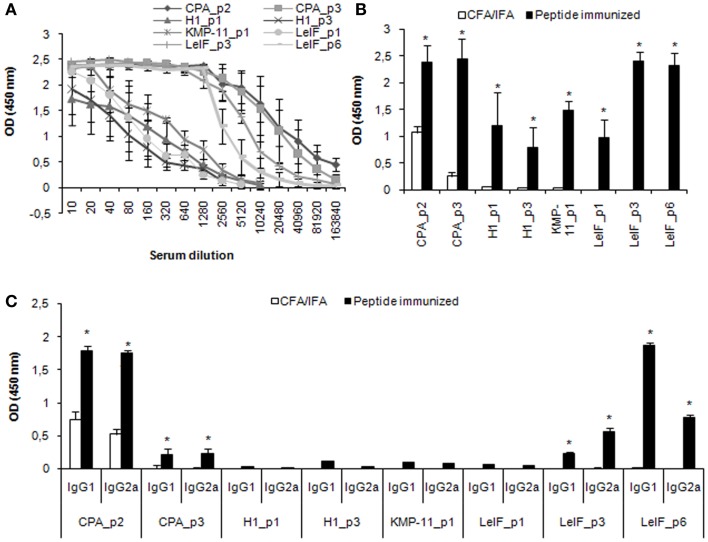
**Multi-epitope peptide-specific antibody production**. BALB/c mice (*n* = 9/group) immunized either with individual peptide emulsified in CFA/IFA or PBS alone, were bled 15 days post third immunization and sera were separated. **(A,B)** total IgG Abs, and **(C)** IgG1 and IgG2a Abs against each peptide were assessed by ELISA. The results are expressed as OD_450_ ± SD. Significant differences between groups of mice immunized with each synthetic peptide emulsified in CFA/IFA and the group of mice immunized with CFA/IFA alone are indicated by * (*P* < 0.05).

Furthermore, specific antibodies of IgG class, as well as of IgG1 and IgG2a isotypes, were detected in the serum of mice immunized with each synthetic peptide emulsified in CFA/IFA, 15 days post third immunization, in order to evaluate peptide effect on humoral response. According to the results, all the synthetic multi-epitope peptides were able to induce the secretion of specific IgG antibodies (Figures 3A,B). Of these, CPA_p2, CPA_p3, LeIF_p3, and LeIF_p6 induced the highest secretion, followed by KMP-11_p1, LeIF_p1, H1_p1, and H1_p3. Analysis of isotype pattern showed that CPA_p2 strongly induced the production of both IgG2a and IgG1 isotypes, followed by CPA_p3, while LeIF_p3 induced the production of IgG2a > IgG1 (Figure 3C). In contrast, LeIF_p6 strongly induced the production of IgG1 isotype and weakly the production of IgG2a isotype. The other four peptides, KMP-11_p1, LeIF_p1, H1_p1, and H1_p3, had insignificant effect on the production of these two IgG isotypes.

**Figure 4:**
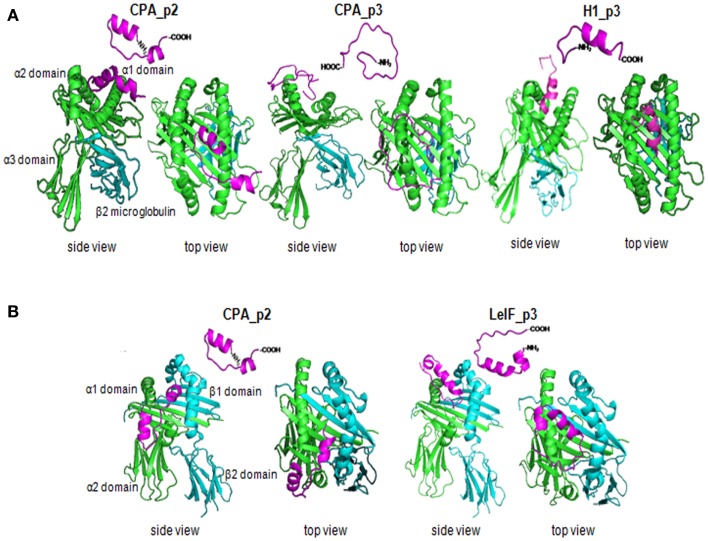
**Synthetic multi-epitope peptides docking**. Ribbon diagram of 3D structural analysis of interactions between **(A)** HLA-A2 molecule (PDB-code: 1HHG) and the synthetic peptides CPA_p2, CPA_p3, H1_p3, and LeIF_p3, and **(B)** HLA-DRB1 molecule (PDB-code: 2SEB) and the synthetic peptides CPA_p2 and LeIF_p3. Candidate peptides were predicted to locate onto the peptide-binding cleft of the HLA molecules by using ClusPro program. The side and top view are shown, the α strands were shown in green, the β strands in blue, and the multi-epitope peptides in magenta.

Next, we employed a structure-based method for further analysis of the tertiary structure of the most promising synthetic peptides (CPA_p2, CPA_p3, H1_p3, LeIF_p3) that bound to HLA-A2 or HLA-DRB1 molecule, since HLA-restricted epitopes were also nested in peptide sequences according to algorithms prediction (Table 4). Selection of HLA-A2 and HLA-DRB1 molecules was based on published data demonstrating high frequency of these supertypes in human population (54, 55). The ClusPro program was run to predict docked conformations presenting good surface complementarity with the two MHC molecules mentioned above. The most probable 3D models according to algorithm analysis indicating peptides located onto the peptide-binding cleft of the MHC molecules with good surface complementarity are presented in Figure 4.

## Discussion

In the perspective of second generation vaccines, a variety of different parasite molecules, such as secretory or transmembrane proteins, including enzymes and receptors, has been tested to date as candidate antigens for anti-*Leishmania* vaccine development (56). Among them, CPA, Histone H1, KMP-11, and LeIF were found to be highly immunogenic as described in murine experimental models, cured VL patients and *L. infantum* infected dogs and have been considered as potential vaccine candidates (39–41, 44, 45, 57). The induction of an effective T cell response against vaccine antigens requires antigen processing and peptide presentation by antigen-presenting cells (APCs), and it is well-established that T cells recognize the peptide sequence in association to appropriate MHC molecules. The discovery of MHC-binding motifs in proteins has led to the development of several algorithms predicting MHC class I- and II-restricted epitopes for presentation to CD8^+^ or CD4^+^ T cells, respectively, accelerating research related to peptide-based vaccine approach (15).

In the present study, we investigated the use of three algorithms, SYFPEITHI, BIMAS, and NetMHCII to predict sequences in CPA, Histone H1, KMP-11, and LeIF able to bind to MHC class I and II molecules of the H2^d^ haplotype. Furthermore, combining this approach with experimental validation in MHC compatible BALB/c mice, we determined epitopes in each protein and designed multi-epitope peptides capable to induce peptide-specific T cell proliferation and cytokine production by CD4^+^ and/or CD8^+^ T cells.

The analysis of protein sequences yielded a significant number of possible epitopes from all four proteins, but only few of them were predicted by all algorithms used with binding efficiency to more than one supertypes or alleles. Interestingly, comparison of predicted peptides for each MHC class I and II alleles showed a low overlapping level between the results obtained from different algorithms used in the study, indicating the significant differences existing in the database source of building matrix motifs and different forms of scoring function of each algorithm. Also, it was observed an antigenic region clustering. Based on these findings and to the fact that prediction of MHC class I-restricted epitopes is considered more reliable (>85%) than that of MHC class II-restricted epitopes, we designed eight multi-epitope peptides for all proteins, based predominantly on highly scored MHC class I-restricted epitopes. Adjacent or overlapping MHC class II-restricted epitopes scored high were also nested in each synthetic peptide. These multi-epitope peptides contained epitopes recognized also by HLA class I and II molecules as defined by *in silico* analysis. Until now, very few vaccine antigens against different pathogens such as viruses, bacteria, and parasites, contain promiscuous T cell epitopes that have the ability to induce T cell-mediated protective immune responses both in mice and human by binding to several alleles of a supertype or between different supertypes (58, 59). Thus, these promiscuous epitope-driven vaccines could have the capacity of increasing the frequency of responders in genetically variable species, such as human populations (60).

The success of many vaccines is dependent on IFNγ-secreting CD4^+^ T cells recruitment for long term protection. This accounts for better immunologic memory leading to sustained immunity after healing of live infections (61, 62). CD4^+^ T cells are activated in terms of recognition of peptides-MHC class II complexes in the surface of APCs after protein processing in cells’ endocytic compartment. Activation of IFN-γ-producing CD4^+^ T cells plays a pivotal role in protective immune responses against leishmaniasis. Specifically, IFN-γ mediates macrophage activation against both the promastigote and amastigote forms in H_2_O_2_-dependent manner (63, 64) and nitric oxide production for parasite killing (65). According to our results, CPA_p2, CPA_p3, LeIF_p3, and LeIF_p6 induced peptide-specific IFN-γ production from CD4^+^ T cells in immunized mice indicating the processing and recognition of MHC class II-specific epitopes by CD4^+^ T cells. CPA and LeIF are considered significant candidate proteins for vaccine design against leishmaniasis. In the case of CPA, it has been shown that administration of plasmid encoding CPA induced specific T_H1_ immune responses resulting to partial protection against *L. major* in the experimental model of CL. However, protection was significantly enhanced when co-administered with CPB or as CPA/B hybrid protein (25–27), indicating the need of CPA co-administration with another protein or adjuvant. On the other hand, LeIF was originally described as a T_H1_-type natural adjuvant and as an antigen inducing an IL-12 mediated T_H1_ response in the PBMCs of leishmaniasis patients (39). LeIF is also capable of inducing the secretion of cytokines IL-12, IL-10, and TNF-α by APCs from healthy individuals (52, 66, 67). Furthermore, recombinant trifusion vaccines (leish111; leish110f) were developed by incorporating the amino-terminal region of LeIF antigen. These vaccines were shown to be efficient in experimental or clinical trials for vaccination or immunotherapy (68).

Existing data suggest that secretory and surface exposed proteins strongly induce specific CD8^+^ T cell responses (69, 70). A previous study applying *in silico* analysis revealed that a high number of peptides derived from *L. major* secretome could bind to H2 BALB/c molecules (71). Several studies have shown the great role played by CD8^+^ T cells in protective immune responses against parasite in the susceptible BALB/c strain (72–74). Specifically, CD8^+^ T cells either contributed in the destruction of *Leishmania*-infected cells by activating macrophages to oxidative burst via cytokines produced upon antigen stimulation (75, 76), or regulating CD4^+^ T cell-mediated immune responses (77, 78). In our study, both synthetic multi-epitope peptides of CPA, CPA_p2, and CPA_p3, except from CD4^+^IFN-γ^+^ T cells activation, induced significant IFN-γ production by CD8^+^ T cells. CPA_p2 and CPA_p3 belong to the secreted region of CPA as SignalP analysis showed (30, 31). Interestingly, another study applying *in silico* analysis in CPA sequence with MULTIPRED algorithm indicated the existence of four highly immunogenic regions recognized by the HLA-A2 supertype (79), which harbored parts from our CPA multi-epitope peptides.

As for KMP-11, KMP-11_p1 belonged to the amino-terminal region of the protein and in contrast to previous observations this synthetic peptide was proved to be poorly immunogenic, indicated by the absence of peptide-specific proliferative response and cytokine secretion in immunized mice. Previous studies concerning the identification of T cell epitopes using infected macrophages or DCs as APCs, revealed the existence of potential HLA class I- and II-restricted T cell epitopes in the amino-terminal region, characterizing a dominant cluster between position 1 and 33 of KMP-11 sequence that could trigger specific cellular immune responses in *L. donovani*- or *L. panamensis*-infected volunteers (80, 81). Furthermore, hybrid-cell, DNA-based or heterologous KMP-11-DNA/rVV based vaccination exhibited immunoprotective capacity in susceptible VL murine models. Protection was accompanied with generation of antigen specific CD4^+^ and CD8^+^ T cells that produced effector cytokines such as IFN-γ, IL-2, and TNF-α (36–38, 82, 83). Also in a previous work, we demonstrated that vaccination with *ex vivo* pulsed bone marrow-derived dendritic cells with KMP-11_12–31aa_ peptide and CpG as adjuvant induced strong Th1 and Th17 protective immune responses in murine model of VL (50). However, in the present study it is noteworthy that secretion of IL-10 was also abrogated. These results together suggest that KMP-11_p1 may be consisted from natural epitopes contributing in parasite host immunomodulation, allowing parasite dissemination rather than stimulate protective immune responses.

However, not only external or secreted *Leishmania* antigens are able to be presented in the context of MHC class I molecules but also intracellular proteins (84, 85). As such, in our study H1_p1 and H1_p3 induced a T cell response characterized mainly by CD8^+^ T cell priming and production of IFN-γ in immunized mice. Although, this way of cell activation in leishmaniasis remains controversial and it is not clear how non-secretory parasite antigens such as histone H1 can be presented endogenously in the context of MHC class I molecules, a number of studies supports the induction of specific CD8^+^ T cell responses against structural parasite proteins in animal models and VL patients (82, 84, 86, 87). Previous results from our group supported that *ex vivo* pulsed bone marrow-derived dendritic cells with the *Leishmania* histone H1 elicited significant protection in the experimental model of VL, with a pronounced enhancement of parasite-specific IFNγ-producing CD8^+^ T cells (88). The protective effect of *Leishmania* histone H1 against *L. major* or *L. infantum* infections was also shown in different experimental animal models (34, 35) suggesting that it is also a promising vaccine candidate against leishmaniasis. In contrast, none of the LeIF peptides tested could evoke specific CD8^+^ T cell responses. This finding was in agreement with the study of Rafati et al. showing that PBMCs from patients recovered from *L. major* failed to elicit HLA-A2-restricted CD8^+^ T cell responses against three synthetic nonamer peptides of LeIF, suggesting that these peptides are not able to induce a CD8^+^ T cell-induced protective immunity (86).

The relative low concentrations of IL-10 detected in the supernatants of immune lymphocytes compared to IFN-γ after peptide re-stimulation were consistent with the suggestion of a dynamic reciprocal relationship between these two cytokines. IL-10 primarily down-modulates innate as well as acquired immunity leading to parasite establishment or disease progression. In experimental model of VL, IL-10 prevents DCs migration in spleen to activate T cells (89, 90) and suppresses both T_H1_ and T_H2_ cells (91). Also, the CD4^+^IL-4^+^ T lymphocytes detected in the presence of all peptides may be attributed to BALB/c intrinsic feature to induce the production of type-2 cytokines, such as IL-4 (53), since there was not any significant difference of IL-4 levels between peptide-immunized mice and control mice receiving the adjuvant alone. Furthermore, IFA adjuvant has a propensity to induce preferentially T_H2_ cytokines (92, 93). Similar study for the evaluation of immunoreactivity of *in silico* predicted T_H1_ epitopes of *Schistosoma japonicum* showed that high levels of IL-4 were attributed to Freund’s adjuvant and BALB/c strain used (94). Therefore, in terms of proportion of intracellular cytokine production of recall CD8^+^ and CD4^+^ T cells, it is concluded that CPA_p3, H1_p1, H1_p3, CPA_p2, LeIF_p3, and LeIF_p6 synthetic multi-epitope peptides are likely to include potential epitopes for the induction of protective cytotoxic (CTL) and T_H1_-type immune responses. Taken into account that the sequences of these synthetic peptides are highly conserved and bind in a promiscuous manner to murine or human MHC molecules according to *in silico* analysis and structure-based techniques, make them candidate vaccines against leishmaniasis. Based on these results, it would be worthwhile conducting future investigations for the verification of peptides’ possible ability to induce protection in common or humanized mouse models of leishmaniasis. The incorporation of alternative and/or additional epitopes, the use of modern adjuvants and new antigen delivery systems should be combined. Conclusively, these findings give complementary data on epitope mapping for *Leishmania* proteins and demonstrate that combination of immunoinformatic approaches with experimental validation enables peptide identification with greater accuracy contributing to rational epitope-based vaccine development.

## Author Contributions

Evita Athanasiou and Olga Koutsoni contributed equally to this work. Conceived and designed the experiments: Evdokia Karagouni. Performed computational analysis: Maria Agallou, Evita Athanasiou, Olga Koutsoni, Evdokia Karagouni. Performed the experiments: Maria Agallou, Olga Koutsoni, Evita Athanasiou. Analyzed the data: Maria Agallou, Evita Athanasiou, Olga Koutsoni, Evdokia Karagouni, Eleni Dotsika. Wrote the paper: Evdokia Karagouni, Maria Agallou.

## Conflict of Interest Statement

The authors declare that the research was conducted in the absence of any commercial or financial relationships that could be construed as a potential conflict of interest.

## Footnotes

^1^http://www.who.int/en/

^2^http://www.ncbi.nlm.nih.gov/genbank

^3^http://www.cbs.dtu.dk/services/SignalP

^4^http://www.syfpeithi.de

^5^http://www-bimas.cit.nih.gov/molbio/hla_bind

^6^http://www.cbs.dtu.dk/services/NetMHCII

^7^blast.ncbi.nlm.nih.gov/Blast.cgi

^8^http://cluspro.bu.edu
